# Impact evaluation of Zika epidemic on congenital anomalies registration in Brazil: An interrupted time series analysis

**DOI:** 10.1371/journal.pntd.0007721

**Published:** 2019-09-23

**Authors:** Enny S. Paixão, Moreno S. Rodrigues, Luciana L. Cardim, Juliane F. Oliveira, Catharina L. C., Maria da Conceição N. Costa, Maurício L. Barreto, Laura C. Rodrigues, Liam Smeeth, Roberto F. S. Andrade, Wanderson K. Oliveira, Maria Glória Teixeira

**Affiliations:** 1 Centro de Integração de Dados e Conhecimentos para Saúde, Centro de Pesquisas Gonçalo Muniz, Fiocruz, Salvador,Brazil; 2 Epidemiology and Population Health, London School of Hygiene and Tropical Medicine, London, United Kingdom; 3 Instituto de Saúde Coletiva, Universidade Federal da Bahia, Salvador, Bahia, Brazil; 4 Instituto de Física, Universidade Federal da Bahia, Salvador, Bahia, Brazil; 5 Secretaria de Vigilância em Saúde, Ministério da Saúde, Brasília, Distrito Federal, Brazil; University of Queensland, AUSTRALIA

## Abstract

This study aimed to assess the impact of the Zika epidemic on the registration of birth defects in Brazil. We used an interrupted time series analysis design to identify changes in the trends in the registration of congenital anomalies. We obtained monthly data from Brazilian Live Birth Information System and used two outcome definitions: 1) rate of congenital malformation of the brain and eye (likely to be affected by Zika and its complications) 2) rate of congenital malformation not related to the brain or eye unlikely to be causally affected by Zika. The period between maternal infection with Zika and diagnosis of congenital abnormality attributable to the infection is around six months. We therefore used September 2015 as the interruption point in the time series, six months following March 2015 when cases of Zika started to increase. For the purposes of this analysis, we considered the period from January 2010 to September 2015 to be “pre-Zika event,” and the period from just after September 2015 to December 2017 to be “post-Zika event.” We found that immediately after the interruption point, there was a great increase in the notification rate of congenital anomalies of 14.9/10,000 live births in the brain and eye group and of 5.2/10,000 live births in the group not related with brain or eye malformations. This increase in reporting was in all regions of the country (except in the South) and especially in the Northeast. In the period “post-Zika event”, unlike the brain and eye group which showed a monthly decrease, the group without brain or eye malformations showed a slow but significant increase (relative to the pre-Zika trend) of 0.2/10,000 live births. These findings suggest an overall improvement in the registration of birth malformations, including malformations that were not attributed to Zika, during and after the Zika epidemic.

## Author summary

Zika can be characterized as one of the most significant emerging arboviruses. The Zika epidemic in Brazil and the subsequent increase in the number of serious brain anomalies, such as microcephaly, has demonstrated the importance of analysing the impact of Zika infection on the rate of congenital anomalies in an affected population. From the analysis of the monthly data on the Live Birth Information System, the authors found that immediately after the Zika event there was a large increase in the notification rate of congenital anomalies reported as a complication of which infection (malformations of brain and eye) and also an increase in the rate of congenital malformations not related with Zika. This growth was seen throughout the country as a whole and in all regions (except in the South), especially in the Northeast where the infection rates were the highest. In the period post-Zika event, the group not related with brain or eye malformation there was an increase in the monthly notification rate while in the brain and eye group there was a decrease in the monthly notification rate. These findings suggest an overall growing awareness of health professionals to improve the registration of birth malformations trigged by the Zika epidemic.

## Introduction

Zika is a vector-borne disease that has become an important concern for the global health agenda after the World Health Organization (WHO) declared a public health emergency of international concern (PHEIC) when it was associated with an epidemic of severe microcephaly cases[1–3].

Ever since the causal relationship between prenatal Zika virus (ZIKV) infection and microcephaly (among other serious brain anomalies) has been established [4]. One important research question is the impact of Zika infection on the rate of congenital anomalies in an affected population. However, this question has proved to be difficult to answer in Brazil and maybe in other Zika affected countries. In Brazil, the main source of information on birth anomalies is the Live Birth Information System (SINASC) that is well known for underreporting cases of congenital anomalies[5–9].

Surveillance data on congenital anomalies is an attractive source of information due to its universality (it covers more than 98% of live births in Brazil, for example) [10,11]. However, the surveillance system that relies on passive case finding strategies, such as SINASC, may be more susceptible to underreporting[12–14]. The rate of under estimation observed in SINASC varies from 36% to 47% in general, but in categories such as microcephaly, this rate be as high as 75% [7].

In 2015, a series of events, triggered by the Zika epidemic, had great potential to change the practices that impact the registration of congenital anomalies. These events were: the alarming growth in suspected cases of a rare condition (microcephaly); measures adopted to strengthen surveillance systems of congenital anomalies in regions where Zika cases had been reported; and finally the massive media coverage on the birth of babies with small heads[15]. Despite the well-known rise in the rate of congenital anomalies related with Zika complications during the epidemic, little has been described on whether some changes in the patterns of recording congenital anomalies not related to Zika have occurred over time. Therefore, this study aims to assess the impact of Zika epidemic on the registration of congenital anomalies in infants in Brazil.

## Methods

We used an interrupted time series analysis (ITSA) design to identify changes in the trends of the registration of congenital anomalies in the country and its regions before and during the Zika epidemic from 2010 to 2017.

### Data source and study population

In this study we used the data from Brazilian live birth information system (SINASC). This system is updated with the registration of a live birth. The system uses a legal document, created in 1990 and compulsorily used throughout the country. The forms are pre-numbered and in three copies are identified by colours (white—the form kept by the local health council that digitizes the information and sends it to the Brazilian Information System headquarters; yellow—kept by the local registry office that generates a birth certificate; pink—kept with the health records of the pregnant women or the neonate in the facility). Mothers do not have to consent to the registration. Data available on this system are collected in a standard form which is completed by the health professional who assisted the delivery, mostly physicians as more than 98% of deliveries take place in hospital. SINASC includes information on the newborn (sex; birth weight, presence of abnormality), the mother (name, place of residence, age, marital status, education) and the pregnancy (length of gestation, type of delivery). Congenital anomalies observed at birth must be described using the International Classification of Diseases (ICD-10) 10th revision. In case of doubt about the condition, paediatricians or neonatologists should be contacted. If none of these professionals were available in the institution the SINASC headquarters must be contacted. These data have a high completeness rate, with missing data not exceeding 10% of most variables[16]. An evaluation of the birth registration system in Brazil found that 98% of Brazilian live births are registered in the system, although some difference are found within regions. However, it should be noted that all regions reach percentages of coverage over 90%[17].

### Procedures

We downloaded the SINASC files on live birth information registered in the period January 2010 to December 2017. We selected seven variables: (i) maternal age, (ii) maternal place of residence, (iii) presence of congenital malformation in the newborn (iv) malformation diagnosis according to International Classification of Diseases, 10th Revision (ICD-10), (vi) newborn date of birth (vii) newborn sex.

We divided our outcome in two categories:1) the rate of congenital malformation of the brain and eye coded as Q00-Q04 and Q10-Q15 in (ICD-10) per 10.000; 2) the rate of congenital malformation not related to the brain or eye, coded as Q05-Q07, Q16-Q18, Q20-Q28, Q30-Q34, Q35-Q37, Q38-Q45, Q50-Q56, Q60-Q64, Q65-Q79, Q80-Q89, Q90-Q99 in ICD-10 per 10,000. We separated the outcomes in these two categories because after the identification of Zika related abnormalities, the first group of ICD-10 codes were potentially related to ZIKV and its complications[18].

The event analysed in this study was the Zika epidemic in Brazil. The design of this study is an interrupted time series because the “event analysed” is expected to “interrupt” the level and/or trend of the outcome variable after its introduction. However, because we are analysing events occurring at birth, we expected a delay in the outcome after maternal exposure during pregnancy. Therefore we considered that: at the beginning of the Zika epidemic, cases were not compulsorily notifiable (the Brazilian surveillance system was not able to record all the disease cases systematically), and there was a delay of about six months between exposure (maternal infection) and outcome (live birth with congenital abnormalities). We therefore used the following approach to select the interruption point in the time series. Firstly we searched in the literature, and considered published studies that estimated the rise of Zika infections cases or exanthematous illness related to Zika. The rise in cases was reported to have started in March 2015 [1, 19]. We implemented a delay of six months from the month when the number of cases started to increase and therefore used September 2015 as the interruption point in the time series. For the purposes of this analysis, we considered the period from January 2010 to September 2015 to be “pre-Zika event,” and the period from just after September 2015 to December 2017 to be “post-Zika event.”

### Statistical analysis

To summarize the characteristics of congenital anomalies according to newborn, sex, maternal age and ICD-10 diagnosis categories, we used descriptive statistics. To assess the impact of Zika epidemic on registry of congenital anomalies, we used an Interrupted Time-series Analysis (ITSA) for a single group. ITSA model for a single group (i.e. a single time series) assumes the following form:

$$y_{t}=\beta_{0}+\beta_{1}T_{t}+\beta_{2}X_{t}+\beta_{3}X_{t}T_{t}+\epsilon_{t},$$

Where y_t_ is the number of cases of malformation divided by the number of births multiplied by 100000 (rate) in each month; T_t_ is the time since the start of the study; X_t_ is a dummy variable that was 0 if the birth date was before Sept 2015 or 1 otherwise; X_t_T_t_ is the interaction between the time and the dummy variable[20]. We use this model to estimate four parameters: (i) *β*_*0*_ that is the rate of malformation at T_0_ (“Pre-zika starting level”), (ii) *β*_*1*_ the mean increase in the malformation rate monthly before Sept 2015 (“Pre Zika event”), (iii) *β*_*2*_ is a change in the slope after Sept 2015 (immediately change) and (iv) *β*_*3*_ the mean increase in malformation rate after Sept 2015 (“Post Zika event”). Furthermore, for each β estimated in our model a t-test is performed to check the parameter values is equal to 0. We assumed that any time-varying unmeasured confounder is relatively slowly changing so that it would be distinguishable from the sharp jump of the event indicator (Zika epidemic).

We performed the ITSA for each of the groups defined in the data preparation section. We did our calculations using Stata SE version 15.

### Ethics statements

We obtained ethical approval from the Federal University of Bahia research ethics committee, Salvador, Brazil (CAAE: 70745617.2.0000.5030). All the data analysed were anonymized.

## Results

A total of 141,969 (0.6% of 23,359,499 live births) congenital abnormalities were registered in SINASC from 2010 to 2017. In Brazil, the starting rate of overall congenital malformations was estimated at 77.2/10,000 live births varying from 56/10,000 live births in the North to 89.3/10,000 live births in Southeast. The distribution of congenital anomalies by ICD-10 group varied over the years, mainly in the number of malformations of the nervous system that peaked in 2016; rates of malformations of the eye, ear, face and neck and malformations of the circulatory system increased over the years from 6.5% in 2010 to 9.2% and 7.2% in 2010 to 11.1% in 2017 respectively. Reporting of other congenital malformations has slowly decreased over time from 6.8% in 2010 to 5.3% in 2017. Maternal age and newborn sex distributions remained similar over the years, although the proportion of babies with congenital anomalies who were born to women over the age of 35 increased from 13.4% to 16.9%. (see Table 1).

**Table 1:** Characteristics of live birth with congenital anomalies in Brazil, 2010–2017.

	2010		2011		2012		2013		2014		2015		2016		2017	
	n	%	n	%	n	%	n	%	n	%	n	%	n	%	n	%
**Newborn sex**																
Male	8,968	55.30	9,765	55.76	9,789	57.12	9,904	57.11	9,493	55.76	10,343	56.06	10,902	56.59	10,813	56.86
Female	6,972	42.99	7,475	42.68	7,349	42.88	7,439	42.89	7,228	42.45	8,106	43.94	8,363	43.41	8,204	43.14
Undetermined	277	1.71	274	1.56	0	0.00	0	0.00	305	1.79	0	0.00	0	0.00	0	0.00
**Age of the mother**																
< 20 years	3,090	19.05	3,389	19.35	3,367	19.65	3,376	19.47	3,167	18.60	3,399	18.42	3,386	17.58	3,037	15.97
20–35 years	10,950	67.52	11,767	67.19	11,396	66.50	11,456	66.06	11,346	66.64	12,201	66.13	12,816	66.52	12,767	67.13
≥ 35 years	2,177	13.42	2,358	13.46	2,375	13.86	2,511	14.48	2,513	14.76	2,849	15.44	3,063	15.90	3,213	16.90
**ICD-10 code**																
Congenital malformations of the nervous system	2,108	13.00	2,277	13.00	2,179	12.71	2,162	12.47	2,203	12.94	2,945	15.96	3,331	17.29	2,587	13.60
Congenital malformations of the eye, ear, face and neck	1,051	6.48	1,264	7.22	1,365	7.96	1,433	8.26	1,369	8.04	1,647	8.93	1,678	8.71	1,754	9.22
Congenital malformations of the circulatory system	1,164	7.18	1,402	8.01	1,541	8.99	1,748	10.08	1,646	9.67	1,696	9.19	1,879	9.75	2,106	11.07
Congenital malformations of the respiratory tract	192	1.18	287	1.64	265	1.55	311	1.79	305	1.79	272	1.47	265	1.38	314	1.65
Lip cleft and cleft palate	1,266	7.81	1,391	7.94	1,297	7.57	1,343	7.74	1,297	7.62	1,341	7.27	1,375	7.14	1,369	7.20
Other congenital malformations of the digestive tract	876	5.40	1,018	5.81	891	5.20	940	5.42	897	5.27	927	5.02	945	4.91	1,008	5.30
Congenital malformations of the genitals	1,220	7.52	1,221	6.97	1,253	7.31	1,241	7.16	1,264	7.42	1,291	7.00	1,329	6.90	1,414	7.44
Congenital malformations of the urinary tract	363	2.24	413	2.36	416	2.43	379	2.19	375	2.20	399	2.16	388	2.01	435	2.29
Congenital malformations of the musculoskeletal system	6,103	37.63	6,262	35.75	6,141	35.83	6,061	34.95	6,025	35.39	6,154	33.36	6,379	33.11	6,317	33.22
Other congenital malformations	1,096	6.76	1,231	7.03	1,13	6.59	1,039	5.99	1,024	6.01	1,080	5.85	1,014	5.26	1,007	5.30
Chromosomal abnormalities, not elsewhere classified	778	4.80	748	4.27	660	3.85	686	3.96	621	3.65	697	3.78	682	3.54	706	3.71

**Fig 1:**
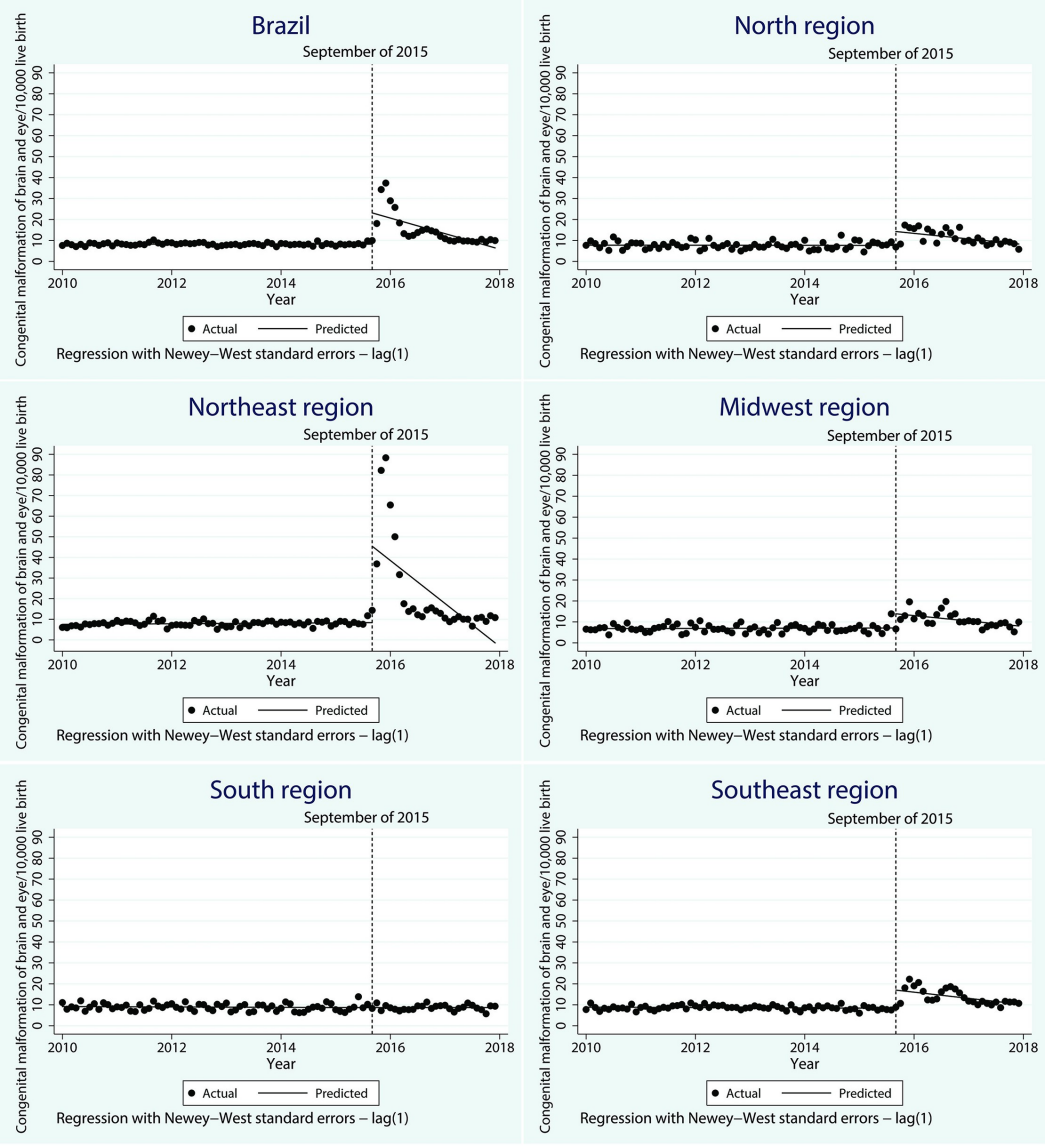
Time series of brain and eye congenital anomalies in Brazil and regions from 2010–2017.

Changes in the rates of reporting congenital malformation rates of the brain and eye are shown in Fig 1 and Table 2. In Brazil, the starting level of congenital malformation rate of the brain and eye was estimated at 8.2/10,000 live births. It was decreasing slowly monthly in the baseline, but it was not significant. Immediately after the interruption point (Sept 2015), the notification rate rose significantly, by 14.9/10,000 live births (CI 95% 6.7–23.2) per month, followed by a significant decrease in the monthly trend (relative to the pre-Zika trend) of 0.6/10,000 (CI 95% -1.1–0.2).

**Table 2:** Changes in the registration rates of brain and eye anomalies and non-brain or eye anomalies/10,000 live births following the Zika event in Brazil and regions, from 2010–2017.

Group of Anomalies	Brazil	North	Northeast	Midwest	South	Southeast
	Estimate	Estimate	Estimate	Estimate	Estimate	Estimate
	95% CI	95% CI	95% CI	95% CI	95% CI	95% CI
**Brain and eye anomalies**						
Pre-Zika starting level	8.24	7.77	7.53	6.80	9.16	8.87
	[7.92–8.57]	[6.97–8.56]	[6.83–8.23]	[6.00–7.61]	[8.49–9.83]	[8.36–9.39]
Pre-Zika event* monthly change	-0.01	-0.01	0.01	0.01	-0.01	-0.01
	[-0.01–0.01]	[-0.02–0.02]	[-0.00–0.03]	[-0.02–0.03]	[-0.03–0.01]	[-0.02–0.01]
Immediate change	14.92	6.64	37.07	6.78	0.03	8.64
	[6.66–23.18]	[3.02–10.26]	[12.88–61.26]	[3.18–10.37]	[-1.31–1.36]	[4.42–12.87]
Post-Zika event* monthly change	-0.62	-0.22	-1.75	-0.22	0.01	-0.24
	[-1.05 - -0.19]	[-0.40 - -0.03]	[-3.00 - -0.49]	[-0.38 - -0.06]	[-0.06–0.07]	[-0.46 - -0.02]
**Non-brain or eye anomalies**						
Pre-Zika starting level	69.57	48.22	60.58	62.21	79.13	80.44
	[67.66–71.49]	[45.68–50.76]	[57.66–63.51]	[59.16–65.26]	[76.54–81.73]	[77.09–83.79]
Pre-Zika event* monthly change	-0.03	-0.12	-0.03	-0.16	-0.10	0.02
	[-0.08–0.01]	[-0.19 - -0.05]	[-0.09–0.03]	[-0.24 - -0.08]	[-0.17 - -0.03]	[-0.07–0.11]
Immediate change	5.20	4.20	10.35	5.42	-1.60	4.34
	[2.30–8.10]	[0.50–7.90]	[6.07–14.62]	[-0.17–11.01]	[-5.69–2.49]	[-1.09–9.77]
Post-Zika event* monthly change	0.22	0.32	0.03	0.10	0.18	0.38
	[0.08–0.36]	[0.14–0.50]	[-0.20–0.25]	[-0.23–0.42]	[-0.05–0.41]	[0.06–0.69]

The North, Northeast, Midwest and Southeast regions showed similar patterns of change. Immediately after the interruption point, the notification rate rose significantly, followed by a significant decrease in the monthly trend (relative to the pre-Zika trend).The most dramatic change occurred in the Northeast region, where the notification rate of brain and eye anomalies immediately post the Zika event went up by 37.1/10,000 live births (95% CI 12.9–61.3) per month, over four times higher than observed in other regions. In the South region, where the circulation of Zika was low, there were no significant changes neither immediately nor over time post the Zika event.

**Fig 2:**
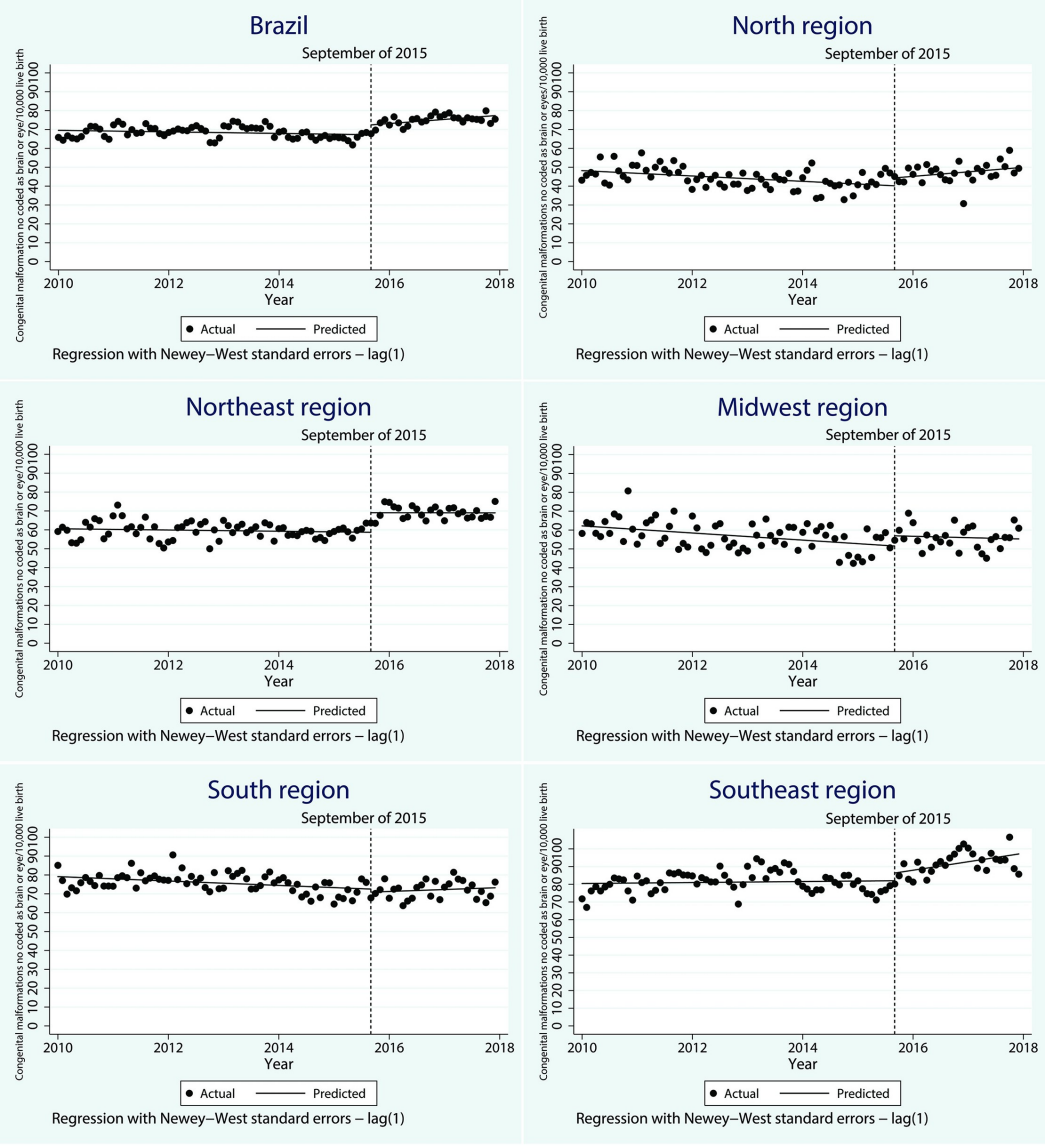
Time series of non-brain or eye congenital anomalies in Brazil and regions from 2010–2017.

As shown in Fig 2 and Table 2, the starting rate of congenital malformations, not coded as brain or eye related, were estimated at 69.6/10,000 live births, and this rate decreased every month prior to Zika by 0.03/10,000 live births (CI 95%-0.08–0.01). However, this was not significant. Right after the interruption point (September 2015), the notification rate of non brain or eye congenital anomalies increased significantly to 5.2/10,000 live births (CI 95% 2.3–8.1), three times lower than observed in the brain and eye group. A slow but significant increase of 0.2/10,000 (CI 95% 0.1–0.4) was observed (relative to the pre-Zika trend) in the monthly notification rates of no brain or eye anomalies.

The North, Northeast, and Southeast regions showed similar patterns of change. An increase in the notification rate of no brain or eye congenital anomalies was observed either right after the interruption point and monthly, however, the effect immediately after the Zika event was not significant in Southeast and the effect over time was not significant in Northeast. In the Midwest and South region, there was no significant change neither immediately nor over time post the Zika event.

We finally conducted a post hoc sensitivity analyses to investigate the earliest month where we got a positive result as an interruption point, and found that March was the first point that the series had broken, it would add to the hypothesis that these findings are Zika-driven.

## Discussion

This study showed that immediately post the Zika event in Brazil, there was a considerable increase in the notification rate of congenital anomalies, mainly malformations of the brain and eye that were reported as a complication of the infection. This growth was reported in all region of the country except in the South, especially in the Northeast, where the majority of Zika cases were concentrated^1^. When the frequency of Zika cases, and consequently risk of maternal infection decreased, the malformations related to its complications also went down significantly, as expected. However, the increased observed (compared with the pre-Zika trends) in the rate of congenital malformations not related to the brain or eye remained at the same level suggesting an overall improvement in the registration of birth malformations. A natural conjecture arises, that such an increase in the registration of cases was in part due to surveillance actions and overall growing awareness of health professionals at the time of the Zika epidemic.

The live birth information system is an attractive source of information on congenital anomalies. Before the circulation of Zika in Brazil the prevalence of congenital anomalies recorded in SINASC was less than 1%, however, it was estimated that the prevalence of congenital anomalies among live births in Brazil was about 2%- 3%[21]. The variation observed across Brazilian regions in the reporting of congenital anomalies rate is possibly due to the heterogeneity of the quality of the notification system, and higher rate of sub registration occurring in the poorest regions of the country. The level of underreporting can also vary by diagnosing groups. A high rate of underreported anomalies has been observed for hydrops, microcephaly, cleft palate, congenital heart disease and Down syndrome[7]. The reported findings suggest that, in part, the increase observed in this study was the result of an active search for cases. Therefore, after the Zika epidemic the live birth information system began to reflect closer to expected levels of notification of birth abnormalities as the reporting system improved.

There are many causes associated with the under-registration of congenital anomalies in the live birth information system, such as uncertainty and delayed diagnosis, deficient knowledge on how to correctly complete the form, and a lack of standardized case definition[22,23]. During the Zika epidemic, the broad press coverage of the malformations resulting from the virus, especially microcephaly, had the effect of changing health care practices and the way cases were recorded. This drew attention to clinical pictures which previously may have been overlooked or incorrectly reported[15]. Improving the quality of medical records of births can lead to a better understanding of the characteristics of children with congenital anomalies, the prevalence of the different types of congenital anomalies and the distribution of these across the country. This can provide crucial information for decision making processes by policy makers.

The great repercussions of the Zika and Congenital Zika Syndrome epidemic may also result in an improvement in prenatal care, either by alerting health professionals to the importance of protocols of care and by making the pregnant women more aware of the importance of pre-natal care and about measures to protect themselves against potentially dangerous infections such as Zika.

In the face of Congenital Zika Syndrome as a result of the Zika epidemic, the overall national emergency response was essential to identify gaps and take steps to strengthen the structure and correct distortions in the registration systems to produce more reliable surveillance systems capable of detecting and notifying cases of birth anomalies. However, after the drop in the number of Zika cases and its complications, there should be concern that some of the operational capacity structured during the epidemic may be dismantled, together with the extra funding and health care resources[24].

Our findings have several limitations. First, there is a lack of knowledge on the spectrum of Congenital Zika Syndrome, therefore in part the excess of cases registered in the no brain or eye ICD-10 group could be explained by unknown Zika complications. Although studies to better understand the spectrum of outcomes associated with maternal ZIKV infection have been developed, and knowledge about the syndrome is improving, the full spectrum of CZS has yet to be defined[25,26]. Secondly, while we have over two years of post-Zika data, it is possible that some effects have not yet become evident. Finally, our study uses routine data that were not specifically created to answer this research question. However, we use the data in high-level aggregate analysis and only use final, rather than provisional data, which are regarded as complete. Despite these limitations this study has provided evidence of improvements in the live birth notification system in registering congenital anomalies triggered by the Zika epidemic.

Congenital anomalies surveillance should be a priority on the public health agenda and CZS has highlighted its importance. Birth defect registration needs to be improved in all developing countries especially now that Zika is also circulating in Africa[27] and Asia[28,29], where birth defect surveillance systems may be even worse than in Brazil.

## Supporting information

S1 File Dataset used in the country level analyses. (DTA)

S2 File Dataset used in the region level analyses. (DTA)
